# Protective effects of paederoside in rotenone-induced cellular models of Parkinson’s disease

**DOI:** 10.3389/fcell.2025.1631652

**Published:** 2025-08-07

**Authors:** Juan Lang, Zhongkui Xiong

**Affiliations:** ^1^ Department of Pathology, Shaoxing People’s Hospital, Shaoxing, Zhejiang, China; ^2^ Department of Medical Imaging, School of Medicine, Shaoxing University, Shaoxing, Zhejiang, China; ^3^ Department of Radiation Oncology, Shaoxing Second Hospital, Shaoxing, Zhejiang, China

**Keywords:** iridoid, Paederia scandens (Lour.), paederoside, Parkinson’s disease, α-synuclein, nitric oxide, nitration, rotenone

## Abstract

**Introduction:**

Parkinson’s disease (PD) is the most prevalent age-related neurodegenerative motor disorder. It affects approximately 1% of individuals aged 65 and older, with its prevalence increasing significantly with advancing age. Current therapeutic approaches primarily focus on symptom management and modestly slowing disease progression, while definitive interventions capable of halting or reversing neurodegeneration remain unavailable. Emerging studies suggest that misfolded proteins progressively accumulate in the neurodegenerating brain, partially attributable to elevated levels of reactive oxygen species and reactive nitrogen species (RNS). The RNS family includes various nitrogen-based compounds, such as nitric oxide (NO), nitroxyl derivatives, and S-nitrosothiol modifications. Phytochemicals have attracted considerable scientific interest as promising candidates for disease-modifying therapies. Prior studies have shown that paederosidic acid, extracted from *P. scandens* (Lour.) Merrill, exhibits notable neuroprotective properties in rodent models. However, the potential of paederoside to confer protection in PD cellular models remains unexplored.

**Methods:**

Paederoside, a bioactive compound isolated from *Paederia* (Rubiaceae family), including *Paederia foetida* and *Paederia scandens*, was evaluated using rotenone-challenged Neuro-2A (N2A) cells and BV-2 microglial cultures, which served as experimental models of PD pathology. Catalpol was used as a comparative pharmacological reference.

**Results:**

In this study, both paederoside and paederosidic acid methyl ester (PAME) significantly reduced NO accumulation in rotenone-induced N2A and BV-2 cells. Paederoside induced a dose-dependent reduction in inducible nitric oxide synthase (iNOS) activity in the rotenone-treated BV-2 cells. When the nuclear factor-κB (NF-κB) inhibitor BAY11-7082 was added 2 h before rotenone exposure, no statistically significant difference in NO levels was observed between the paederoside-treated and untreated groups. Pretreatment with 1 μM or 10 μM of paederoside significantly attenuated the formation of nitrated α-synuclein (α-Syn) in response to rotenone exposure. Furthermore, pretreatment with 10 μM paederoside markedly enhanced cell viability in rotenone-treated N2A cells.

**Discusion:**

In summary, these findings demonstrate the neuroprotective potential of paederoside through modulation of the NF-κB/NOS/NO/nitrated α-Syn nitration signaling pathway.

## 1 Introduction

Parkinson’s disease (PD) is the leading age-associated neurodegenerative motor disorder (Mhyre et al., 2012). This condition affects approximately 1% of individuals over the age of 65, with its prevalence increasing considerably with advancing age (Percario et al., 2020). The etiology of PD involves a complex interplay between environmental factors and genetic predisposition (Dong-Chen et al., 2023). Exposure to neurotoxic agents, in combination with inherited genetic variations, may initiate the cascade of neural degeneration (Dong-Chen et al., 2023). Key pathological features include the progressive loss of dopamine (DA)-producing neurons in the substantia nigra and the presence of α-synuclein (α-Syn)-containing Lewy bodies (LBs) (Bose and Beal, 2016). The protein α-Syn plays a central role in PD pathology through its aberrant structural modifications (Li et al., 2021). Misfolded α-Syn conformations trigger molecular cascades that drive disease progression (Mehra et al., 2019). The pathological accumulation of α-Syn oligomers induces neuronal damage via mitochondrial dysregulation, neuroinflammatory responses, and impaired lysosomal activity (Li et al., 2025). These aggregation phenomena, often associated with redox imbalance, are fundamental mechanisms underlying the neurodegenerative processes of PD (Li et al., 2025).

Emerging studies suggest that misfolded proteins progressively accumulate in the neurodegenerating brain, partially attributable to elevated levels of reactive oxygen species (ROS) and reactive nitrogen species (RNS) (Nakamura et al., 2021). These oxidative agents demonstrate strong pathological correlations with degenerative disorders such as PD (Hoye et al., 2008). The RNS family comprises various nitrogen-based compounds, such as nitric oxide (NO), nitroxyl derivatives, and S-nitrosothiol modifications (Kaur et al., 2022). Both dysregulated NO synthesis and impaired DA homeostasis have been independently implicated in neurodegenerative pathways (Nunes and Laranjinha, 2021). Acting as a key regulator of programmed necrosis, NO promotes nuclear condensation and reduces mitochondrial integrity by activating the phosphatidylinositol phosphate 1-phosphatase–phosphatidylinositol triphosphate–human mixed-lineage kinase-like domain phosphorylation pathway (Zhang et al., 2024). Post-translational modifications mediated by RNS, particularly S-nitrosylation and nitration of tyrosine residues, play critical roles in promoting protein misfolding and pathological aggregation (Nakamura et al., 2021). The detrimental effects of oxidative stress accelerate neurodegeneration through multiple mechanisms (Trist et al., 2019). When neuronal antioxidant defenses are overwhelmed, radical-mediated cytotoxicity induces substantial neuronal damage, ultimately triggering the death of dopaminergic neurons under oxidative duress (Hemmati-Dinarvand et al., 2019).

Current therapeutic strategies primarily focus on alleviating clinical symptoms and decelerating disease progression (Raza et al., 2019). However, no pharmacological breakthroughs have yet succeeded in protecting dopaminergic neurons from degeneration (Raza et al., 2019). Clinically validated approaches capable of arresting or reversing pathological progression remain elusive (Li et al., 2021). Ongoing research predominantly targets the development of low-molecular-weight compounds, peptide-based therapeutics, and aggregation pathway-specific peptidomimetics aimed at α-Syn pathology (Vidovic and Rikalovic, 2022). Furthermore, plant-derived bioactive compounds are increasingly recognized as promising candidates for neuroprotective interventions (Gahtani et al., 2024).

The genus *Paederia* (Rubiaceae) comprises two pharmacologically significant species, *Paederia foetida* and *Paederia scandens*, which are primarily distributed across China, South Asia, and Mauritius. Traditionally, these plants have been used to treat inflammatory conditions, gastrointestinal disorders, and musculoskeletal pain, as documented in ethnopharmacological literature (Wang et al., 2014; Ai et al., 2023; Li et al., 2019). Recent studies indicate that their bioactive components possess therapeutic potential in oncology, inflammation modulation, tissue regeneration, and reproductive health enhancement (Sarma et al., 2023). Experimental data demonstrate that prophylactic administration of *P. foetida* Linn. effectively reduces oxidative stress and cytokine production in models of Freund’s adjuvant-induced inflammation (Kumar et al., 2015). The plant’s foliar extract has exhibited neuroprotective effects in scopolamine-induced cognitive impairment models, suggesting potential application in managing neurodegenerative disorders (Pakaprot et al., 2024). Phytochemical analyses have identified diverse constituents, including iridoid glycosides (asperuloside and paederosidic acid), sterols, phenolic compounds, and triterpenoids within these species (Dwivedi et al., 2025). Notably, iridoids isolated from *P. scandens* exert anti-inflammatory effects through inhibition of the nuclear factor-κB (NF-κB) pathway in macrophage models, thereby suppressing pro-inflammatory mediators such as cyclooxygenase-2 and interleukins (Xu et al., 2023). In studies of renal pathology, methanolic extracts of *P. foetida* exhibit concentration-dependent suppression of NF-kB signaling in diabetic nephropathy models (Borgohain et al., 2017). Pharmacological investigations reveal that administration of iridoid glycosides derived from *P*. *scandens* (IGPS, 70–280 mg/kg) alleviates neuropathic pain in sciatic nerve injury models, accompanied by reductions in nitric oxide synthase (NOS) activity and secondary messenger levels (Liu et al., 2012). Root extracts of *P. scandens* yield specific iridoid derivatives, including paederoside and its acidic counterpart, paederosidic acid (Quang et al., 2002). Preclinical trials with paederosidic acid methyl ester (PAME) indicate analgesic effects mediated through NO–cyclic guanosine monophosphate (cGMP) signaling and potassium channel modulation in murine pain models (Chen et al., 2009).

Prior studies have shown that paederosidic acid, extracted from *P*. *scandens* (Lour.) Merrill, exhibits notable neuroprotective properties in rodent models (Yang et al., 2013). However, the potential of paederoside to confer protection in PD cellular models remains unexplored. In this study, catalpol was used as a positive control to validate the experimental methodology. The primary objective was to evaluate the therapeutic potential of paederoside in rotenone-induced PD cell models and to investigate its mechanistic role in reducing NO production and suppressing protein nitration along the NF-κB/NOS/NO/nitrated α-Syn signaling axis.

## 2 Materials and methods

### 2.1 Reagents

**FIGURE 1:**
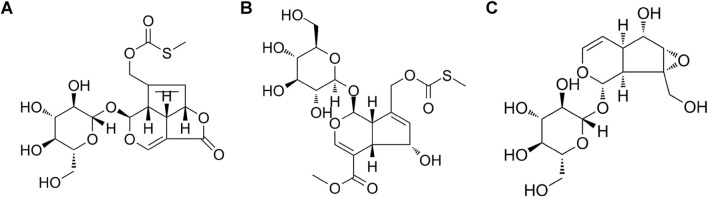
Chemical structure, **(A)** paederoside; **(B)** paederosidic acid methyl ester (PAME); **(C)** catalpol.

Paederoside and PAME were procured from Wuhu Delta Pharmaceutical Technology Co., Ltd., China (Figure 1). Catalpol was obtained from the China Institute for Food and Drug Control (Figure 1). Rotenone and dibutyryl cyclic adenosine monophosphate (db-cAMP) were purchased from Sigma-Aldrich. Dulbecco’s modified Eagle medium (DMEM), minimal essential medium (MEM), fetal bovine serum (FBS), and trypsin were obtained from Invitrogen. The primary mouse monoclonal antibody against human α-Syn (nitrated Tyr125, nitrated Tyr133) was procured from Novus Biologicals. Anti-mouse/rabbit secondary antibodies, the streptavidin–biotin complex (SABC), and 3,3′-diaminobenzidine (DAB) were acquired from Wuhan Boster Biological Technology Co. Ltd., China. The nitric oxide (NO) assay kit was purchased from Abcam. The inducible NOS (iNOS) enzyme-linked immunosorbent assay (ELISA) kit was obtained from EIAab Science Inc., Wuhan, China. NG-monomethyl-L-arginine (L-NMMA), S-methylisothiourea sulfate (SMT), arginine, and BAY11-7082 were purchased from Beyotime Biotechnology, China.

### 2.2 Culture of cell lines

#### 2.2.1 Cell lines

The BV-2 microglial cell line, a murine microglial cell derived from C57BL/6 mice (Ardizzone et al., 2025), was acquired from Shanghai Fuxiang Biotechnology Co., Ltd., China. The Neuron-2A (N2A) cell line, a cell derived from mouse neural crest cells (Tremblay et al., 2010), was sourced from the Cell Bank of the Typical Culture Preservation Committee of the Chinese Academy of Sciences, China.

#### 2.2.2 BV-2 microglial cell culture

BV-2 microglial cells were cultured in DMEM supplemented with 10% FBS, penicillin (50 U/mL), and streptomycin (50 μg/mL) and incubated at 37°C in a humid atmosphere containing 5% CO_2_. Prior to plating, the FBS concentration in the culture medium was reduced to 0.5%. The cell suspension was seeded into 96-well culture plates at 5 × 10^4^ cells/mL density, with 200 μL of cell suspension added to each well.

#### 2.2.3 N2A cell culture and induction of cellular differentiation

Given the unique oncogenic characteristics of N2A cells and their incomplete dopaminergic phenotype, induction of differentiation is necessary for PD modeling. Current differentiation protocols typically involve serum reduction and chemical stimulation. Specifically, the FBS concentration was reduced from 10% to 0.5%, and the medium was supplemented with 1 mM db-cAMP. The optimized differentiation medium consisted of MEM supplementation with 0.5% FBS, 1 mM db-cAMP, penicillin (50 U/mL), and streptomycin (50 μg/mL). Cells were seeded in 96-well culture plates at a density of 5 × 10^4^ cells/mL, with medium replacement and experimental treatments performed on alternating days throughout the study.

### 2.3 Protocol for treatments and examinations

**FIGURE 2:**
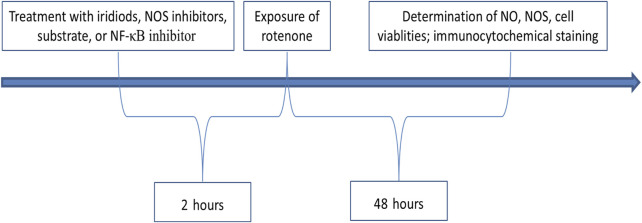
Protocol for treatments and examinations.

As showed in Figure 2, 2 h before exposure of rotenone, N2A or BV-2 cells were treated with iridiods, NOS inhibitors, substrate, or NF-κB inhibitors. Following the addition of rotenone, the cells were cultured for an additional 48 h, after which the following parameters were assessed: NO assay, NOS assay, cell viability assay, and immunocytochemical analysis.

### 2.4 Effect of iridoid compounds on NO levels

#### 2.4.1 Experimental grouping and drug treatment

##### 2.4.1.1 Effect of iridoid compounds on rotenone-induced NO levels in the supernatant of rotenone-exposed cells

This experiment included five groups: control, model, catalpol (positive control), paederoside, and PAME, with the latter three used at a final concentration of 10 μM, 2 h prior to rotenone treatment (Figure 2). Each treatment was performed in triplicate wells and repeated 3–4 times. The sample size (n) refers to the number of independent replicates per experiment. This definition applies throughout the subsequent sections.

##### 2.4.1.2 Dose-dependent effect of paederoside on NO concentrations in the supernatant of rotenone-stimulated cells

Six groups were established: control, model, and four paederoside treatment groups receiving a final concentration of 0.01 μM, 0.1 μM, 1 μM, or 10 μM.

##### 2.4.1.3 Effect of paederoside on NO concentrations in cell supernatants when combined with NOS inhibitors or regulatory agents

This experiment included five groups: control, model, paederoside alone (10 μM), treatment X alone, and treatment X combined with paederoside. X represents one of four possible compounds: L-NMMA (1 mM), a non-specific NOS inhibitor; SMT (1 mM), a selective iNOS inhibitor; L-arginine (1 mM), a substrate for NOS; or BAY11-7082 (5 μM), an NF-κB pathway inhibitor (Figure 2).

In all cases, the regulatory agent and paederoside were co-treated 2 h before rotenone exposure. All experimental conditions adhered to consistent dosing parameters throughout the study.

#### 2.4.2 Determination of NO levels

All laboratory procedures were conducted in accordance with the manufacturer’s instructions provided in the respective assay kits.

### 2.5 Effect of paederoside on iNOS activity

#### 2.5.1 Experimental grouping and drug treatment

##### 2.5.1.1 Effect of paederoside on iNOS activity in rotenone-stimulated BV-2 cell lysates

This experiment examined the effect of paederoside on iNOS levels in BV-2 cells exposed to rotenone. iNOS activity was quantified using ELISA. Five groups were included: control, model, and three treatment groups receiving paederoside at a final concentration of 0.1 μM, 1 μM, or 10 μM.

##### 2.5.1.2 Effect of paederoside on iNOS activity in rotenone-stimulated BV-2 cell lysates when combined with NF-κB pathway suppressor BAY11-7082

This experiment assessed changes in inflammatory mediator expression from co-treatment with a plant bioactive component (paederoside) and a pharmacological inhibitor (BAY11-7082) under neurotoxic (rotenone) conditions. The experimental design included five groups: untreated control, disease model, paederoside alone (10 μM), BAY11-7082 alone (5 μM), and combined paederoside and BAY11-7082. BAY11-7082 and paederoside were co-treated 2 h prior to rotenone exposure.

#### 2.5.2 Measurement of iNOS activity via ELISA

iNOS activity was quantified according to the manufacturer’s instructions provided in the ELISA kit.

### 2.6 Effect of paederoside on nitrated α-syn

#### 2.6.1 N2A/BV-2 mixed cell culture system

N2A and BV-2 cells were co-cultured in 96-well culture plates at a 1:4 seeding ratio. Each well received 200 μL of total cell suspension containing 5 × 10^4^ cells/mL. The culture medium consisted of a 1:1 mixture of DMEM and MEM, supplemented with 0.5% FBS, 0.1 mM db-cAMP, penicillin (50 U/mL), and streptomycin (50 μg/mL).

#### 2.6.2 Experimental grouping and drug treatment

Cells were assigned to five groups: control, model, and three paederoside treatment groups, with the latter receiving a final concentration of 0.1 μM, 1 μM, or 10 μM.

#### 2.6.3 Immunocytochemical analysis

The experimental protocol is as follows (Mhyre et al., 2012). Cells were thawed and allowed to equilibrate to room temperature, followed by three washes with phosphate-buffered saline (PBS) for 5 min each (Percario et al., 2020). Samples were treated with 3% hydrogen peroxide at room temperature for 20 min to quench endogenous peroxidase activity, then washed three times with PBS (Dong-Chen et al., 2023). The cells were then permeabilized using 0.3% Triton X-100 for 30 min at room temperature, followed by three PBS washes (Bose and Beal, 2016). Non-specific binding sites were blocked using 5% bovine serum albumin (BSA) at room temperature for 30 min, followed by three PBS washes (Li et al., 2021). The cells were incubated overnight with mouse anti-human nitrified α-Syn primary antibodies (1:100 dilution) at 4°C, followed by another incubation at 37°C for 60 min. The negative control cells were incubated with 5% BSA alone, followed by three PBS washes (Mehra et al., 2019). The corresponding secondary antibodies (anti-mouse/rabbit) were added dropwise and incubated at 37°C for 60 min, followed by three PBS washes (Li et al., 2025). The SABC was then added dropwise and incubated at room temperature for 30 min, followed by three final PBS washes (Nakamura et al., 2021). DAB chromogenic solution was added under microscopic observation, and the reaction was terminated upon reaching optimal staining intensity for photographic documentation.

### 2.7 Effect of paederoside on cell viability

#### 2.7.1 Experimental grouping and drug treatment

The experiment included five groups: control, model, and three paederoside treatment groups, with the latter receiving 0.1 µM, 1 μM, or 10 µM paederoside. After a 2-h incubation, all groups received 20 nM rotenone and were incubated for an additional 48 h.

#### 2.7.2 Cell viability measurement via CCK-8 assay

At the designated time point, 10 μL of CCK-8 solution was added to each well, followed by incubation for 120 min under 5% CO_2_ at 37°C. Absorbance was measured using a microplate reader at 450 nm, with 620 nm as the reference wavelength. All experiments were performed in triplicate to ensure reproducibility.

### 2.8 Statistical analysis

Data are presented as mean ± standard error of the mean. Statistical analyses were conducted using SAS 6.12 software. One-way ANOVA was used for comparisons among multiple groups. When significant differences were detected, the Student–Newman–Keuls *post hoc* test was applied. Paired Student's *t*-tests were employed for comparison between two groups. A P-value <0.05 was considered statistically significant.

## 3 Results

### 3.1 Effect of iridoid compounds on NO production in rotenone-exposed cell models

#### 3.1.1 Effect of iridoid compounds on NO concentrations in rotenone-exposed N2A cells

**FIGURE 3:**
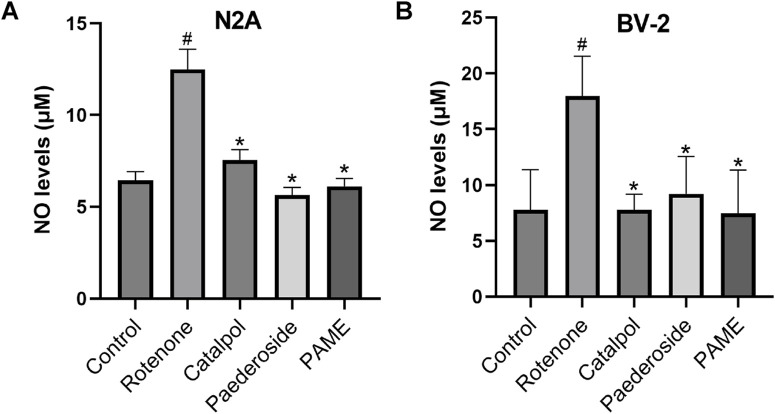
Effect of iridoid compounds on nitric oxide (NO) production in rotenone-exposed N2A **(A)** or BV-2 **(B)** cell models (n = 3). ^#^P < 0.05 vs control group; *P < 0.05 as compared with model group treated with rotenone.

The concentration and timing for the administration of pederoside and PAME were referenced from an investigation into the effects of catalol on a cellular model of PD. Our previous research has found that a concentration of 1 × 10^5^ M of catalpol significantly enhanced the density of TH-positive neurons and the length of neurite outgrowth in cultured mesencephalic neurons after a 48-h exposure to catalpol (Xu et al., 2010). Figure 3A shows NO concentrations measured in the supernatants of the control group, recorded at 6.44 ± 0.48 μM. Following 48-h exposure to 20 nM rotenone, the model group exhibited a 93.8% increase in NO levels (12.48 ± 1.11 μM, P < 0.05 versus controls). A 2-h pretreatment with catalpol, paederoside, and PAME significantly attenuated this elevation, reducing concentrations to 7.55 ± 0.57 μM, 5.64 ± 0.42 μM, and 6.12 ± 0.42 μM, respectively (all P < 0.05 compared to the untreated model group).

#### 3.1.2 Effect of iridoid compounds on NO concentrations in rotenone-exposed BV-2 cells

As shown in Figure 3B, control group supernatants maintained baseline NO concentrations averaging 7.79 ± 1.79 μM. Rotenone exposure (20 nM, 48 h) induced significant NO elevation in the model group, reaching 17.96 ± 1.79 μM, a 130.6% increase relative to controls (P < 0.05). A 2-h pretreatment with catalpol, paederoside, and PAME notably reduced NO levels to 7.79 ± 0.69, 9.22 ± 1.66, and 7.47 ± 1.93 μM, respectively (all P < 0.05 compared to the untreated model group). Comparative analysis revealed comparable efficacies among the three iridoid treatments (P > 0.05), indicating similar pharmacological effects on NO modulation.

### 3.2 Effect of paederoside on NO levels in the supernatant of rotenone-induced cells

#### 3.2.1 Effect of paederoside on NO levels in the supernatant of rotenone-induced N2A cells

**FIGURE 4:**
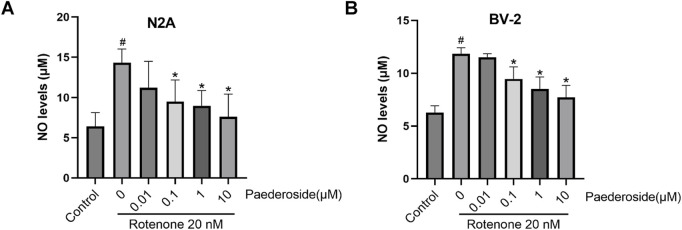
Effect of paederoside on NO levels in the supernatant of rotenone-induced N2A **(A)** or BV-2 **(B)** cells (n = 3). ^#^P < 0.05 vs control group; *P < 0.05 as compared with rotenone-treaed group.

As shown in Figure 4A, the baseline NO concentration was 6.39 ± 0.86 μM in control group supernatants. Rotenone exposure (20 nM, 48 h) elevated NO levels to 14.31 ± 0.86 μM in the model group, representing a 123.9% increase compared to controls (P < 0.05). A 2-h pretreatment with paederoside at 0.1 μM, 1 μM, or 10 μM effectively attenuated this increase, producing dose-dependent reductions to 9.48 ± 1.35 μM, 8.97 ± 0.95 μM, and 7.60 ± 1.41 μM, respectively (all P < 0.05 compared to the model group). Notably, the lowest tested concentration (0.01 μM) showed limited efficacy, yielding NO levels of 11.21 ± 1.64 μM, which were not statistically different from the untreated model group (14.31 ± 0.86 μM) (P > 0.05).

#### 3.2.2 Effect of paederoside on rotenone-induced NO levels in the supernatant of BV-2 cells

As shown in Figure 4B, the baseline NO concentration in control group supernatants was 6.28 ± 0.32 μM. Rotenone exposure (20 nM, 48 h) elevated NO levels to 11.84 ± 0.57 μM, representing an 88.5% increase relative to controls (P < 0.05). A 2-h pretreatment with paederoside at 0.1 μM, 1 μM, and 10 μM resulted in marked reductions to 9.46 ± 0.57 μM, 8.50 ± 0.57 μM, and 7.71 ± 0.57 μM, respectively (P < 0.05 versus model group). Notably, pretreatment with 0.01 μM paederoside maintained NO levels at 11.52 ± 0.16 μM, showing no statistically significant difference compared to the untreated model group (11.84 ± 0.57 μM) (P > 0.05).

### 3.3 Effect of paederoside on iNOS activity in rotenone-induced BV-2 cell lysates

**FIGURE 5:**
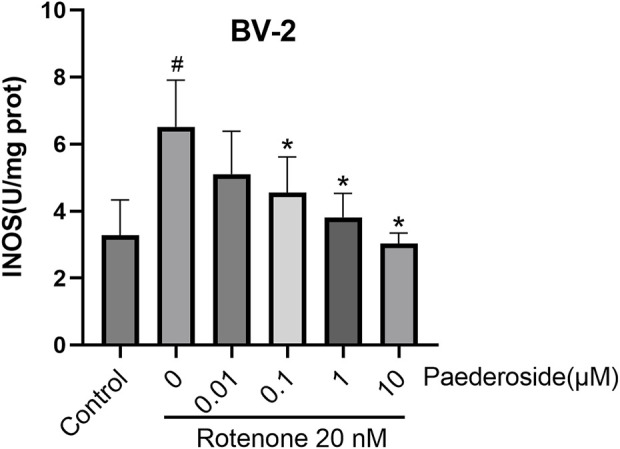
Effect of paederoside on inducible nitric oxide synthase (iNOS) activity in rotenone-induced BV-2 cell lysates (n = 3). ^#^P < 0.05 vs. control group; *P < 0.05 as compared with rotenone group.

Figure 5 shows that iNOS activity in the control group measured 3.27 ± 0.53 U/mg protein, whereas rotenone-exposed cells exhibited a significant increase to 6.51 ± 0.70 U/mg protein, nearly doubling baseline values (a 99.1% increase; P < 0.05). Paederoside treatment induced a dose-dependent decline in iNOS activity, with levels decreasing progressively in the model group from 6.51 ± 0.70 U/mg protein to 4.55 ± 0.92, 3.81 ± 0.36, and 3.02 ± 0.16 U/mg protein (all P < 0.05). The 0.01 μM paederoside dose failed to induce statistically significant changes, maintaining iNOS at 5.10 ± 0.93 U/mg protein compared to 6.51 ± 0.70 U/mg protein in the model group (P > 0.05).

### 3.4 Effect of paederoside on NO levels in rotenone-treated N2A and BV-2 cells under NOS inhibition

#### 3.4.1 Effect of paederoside on NO levels in rotenone-treated N2A cells under NOS inhibition

**FIGURE 6:**
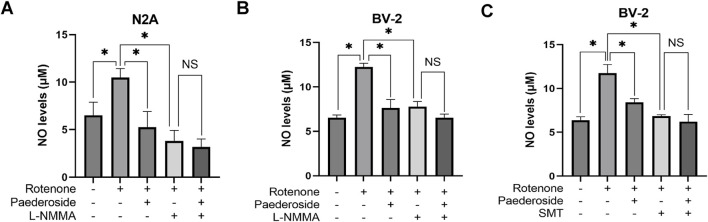
Effect of paederoside on NO levels in rotenone-treated N2A or BV-2 cells under nitric oxide synthase (NOS) inhibition (n = 3). Effect of paederoside on NO levels in rotenone-treated N2A cells under NOS inhibition with L-NMMA **(A)**; Effect of paederoside on NO levels in rotenone-treated BV-2 cells under NOS inhibition with L-NMMA **(B)** or SMT **(C)**. *P < 0.05, ^NS^P > 0.05.

As shown in Figure 6A, untreated controls exhibited NO concentrations of 6.52 ± 0.69 μM in culture supernatants, whereas rotenone-treated samples showed elevated levels of 10.49 ± 0.48 μM, marking a 60.9% increase relative to baseline values (P < 0.05). Treatment with paederoside significantly reduced NO concentrations from 10.49 ± 0.48 μM in the model group to 5.25 ± 1.43 μM, representing a 50.0% suppression of rotenone-induced NO production (P < 0.05). Treatment with L-NMMA also resulted in a significant reduction of NO concentration in rotenone-treated N2A cells (P < 0.05). Co-treatment with the NOS inhibitor L-NMMA with rotenone led to further suppression, lowering NO levels to 3.82 ± 0.55 μM (P < 0.05). Notably, when L-NMMA was pretreated 2 h prior to rotenone, paederoside pretreatment failed to produce statistically significant changes in NO levels (3.18 ± 0.42 μM versus 3.82 ± 0.55 μM, P > 0.05).

#### 3.4.2 Effect of paederoside on NO levels in rotenone-treated BV-2 cells under NOS inhibition

Figure 6B shows that baseline NO concentration was 6.52 ± 0.32 μM in control group supernatants. Rotenone exposure substantially increased to 12.24 ± 0.42 μM, representing an 87.7% surge compared to untreated samples (P < 0.05). Subsequent analysis revealed a 37.7% decrease in NO levels within the model group, dropping from 12.24 ± 0.42 μM to 7.63 ± 0.95 μM (P < 0.05). Treatment with the NOS inhibitor L-NMMA further reduced NO concentrations to 7.79 ± 0.57 μM in the model group (P < 0.05). However, when L-NNMA was added 2 h prior to rotenone, there was no significant difference between paederoside-treated and untreated groups (6.52 ± 0.42 μM versus 7.79 ± 0.57 μM, P > 0.05).

As shown in Figure 6C, supernatant NO levels in untreated controls were 6.36 ± 0.42 μM, whereas rotenone-treated samples showed elevated concentrations of 11.76 ± 0.97 μM, indicating an 84.9% increase from baseline (P < 0.05). Comparative analysis revealed that NO values in the model group decreased from 11.76 ± 0.97 μM in control specimens to 8.43 ± 0.42 μM following treatment, a 28.3% reduction (P < 0.05). Pretreatment with SMT further reduced NO concentrations to 6.84 ± 0.16 μM in the paederoside model groups (P < 0.05). Notably, when paederoside was added following SMT pretreatment, NO levels remained unchanged (6.20 ± 0.83 μM versus 6.84 ± 0.16 μM, P > 0.05), suggesting that paederoside’s protective effect is mediated through modulation of NOS activity, which is inhibited by SMT.

### 3.5 Effect of paederoside on NO levels in rotenone-induced N2A and BV-2 cells in the presence of the NOS substrate arginine

#### 3.5.1 Effect of paederoside on NO levels in rotenone-treated N2A cells under arginine supplementation

**FIGURE 7:**
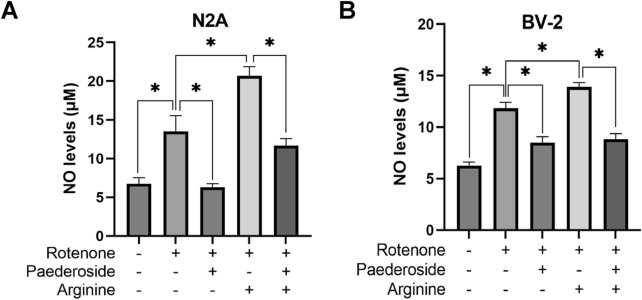
Effect of paederoside on NO levels in rotenone-induced N2A **(A)** and BV-2 **(B)** cells in the presence of the NOS substrate arginine. (n = 3). *P < 0.05.

Figure 7A demonstrates that control group supernatants exhibited NO concentrations of 6.75 ± 0.78 μM, whereas rotenone-treated samples showed elevated levels of 13.50 ± 2.06 μM, indicating a 100% increase relative to controls (P < 0.05). Paederoside treatment effectively reduced NO concentrations from the model group’s baseline of 13.50 ± 2.06 μM to 6.30 ± 0.45 μM, representing a 53.3% decrease (P < 0.05). Supplementation with arginine (NOS substrate) substantially increased NO levels in the model group to 20.69 ± 1.19 μM (P < 0.05). Notably, When co-treated with rotenone and arginine, the intervention of paederoside decreased the NO level to 11.70 ± 0.90 μM (P < 0.05).

#### 3.5.2 Effect of paederoside on NO levels in rotenone-treated BV-2 cells under arginine supplementation

As shown in Figure 7B, the control group supernatants exhibited an NO concentration of 6.28 ± 0.32 μM, while rotenone-treated cells demonstrated an 88.5% increase to 11.84 ± 0.57 μM, compared to controls (P < 0.05). Paederoside treatment significantly reduced NO levels by 28.2%, lowering model group values from 11.84 ± 0.57 μM to 8.50 ± 0.57 μM (P < 0.05). Arginine supplementation elevated NO concentrations to 13.91 ± 0.42 μM in the rotenone-exposed group (P < 0.05), whereas exposure to rotenone 2 h after arginine supplementation paradoxically decreased NO levels to 8.82 ± 0.55 μM (P < 0.05).

### 3.6 Effect of paederoside on NO levels in rotenone-induced cell models in the presence of the NF-κB pathway inhibitor BAY11-7082

#### 3.6.1 Effect of paederoside on NO levels in rotenone-treated N2A cells with NF-κB pathway inhibition by BAY11-7082

**FIGURE 8:**
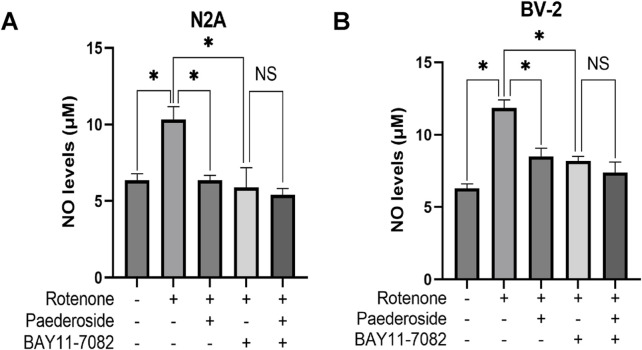
Effect of paederoside on NO levels in rotenone-induced N2A **(A)** and BV-2 **(B)** cell models in the presence of the nuclear factor-κB (NF-κB) pathway inhibitor BAY11-7082 (n = 3). *P < 0.05, ^NS^P > 0.05.

Figure 8A demonstrates that the control group supernatants exhibited an NO concentration of 6.36 ± 0.42 μM, while rotenone-treated cells showed elevated levels of 10.33 ± 0.84 μM, indicating a 62.4% increase relative to controls (P < 0.05). Paederoside treatment reduced NO measurements in the model group from 10.33 ± 0.84 μM to 6.36 ± 0.32 μM, reflecting a 38.4% decrease (P < 0.05). When the NF-κB inhibitor BAY11-7082 was added, NO concentrations in the model group declined further to 5.88 ± 1.30 μM (P < 0.05). In the presence of both BAY11-7082 and rotenone, which were added 2 h post paederoside treatment, no statistically significant difference in NO concentrations was observed between groups with paederoside and without paederoside (5.40 ± 0.42 μM versus 5.88 ± 1.30 μM, P > 0.05).

#### 3.6.2 Effect of paederoside on NO levels in rotenone-treated BV-2 cells with NF-κB pathway inhibition by BAY11-7082

As shown in Figure 8B, control supernatants exhibited NO concentrations of 6.28 ± 0.32 μM, while rotenone-treated cells showed elevated NO levels of 11.84 ± 0.57 μM, representing an 88.5% increase compared to baseline (P < 0.05). Paederoside treatment significantly attenuated this increase, reducing NO levels from 11.84 ± 0.57 μM to 8.50 ± 0.57 μM, equivalent to 28.2% suppression (P < 0.05). Pretreatment with the NF-κB pathway blocker BAY11-7082 2 h prior to rotenone exposure resulted in a decrease to 8.19 ± 0.32 μM (P < 0.05). In the presence of both BAY11-7082 and rotenone, which were added 2 h later, no statistically significant difference in NO concentrations was observed between groups treated with and without paederoside (7.39 ± 0.73 μM versus 8.19 ± 0.32 μM, P > 0.05). These findings suggest that the reduction in NO levels induced by paederoside was attributable to NF-κB inhibition.

### 3.7 Effect of paederoside on iNOS activities in rotenone-induced BV-2 microglial cells under NF-κB pathway inhibition by BAY11-7082

**FIGURE 9:**
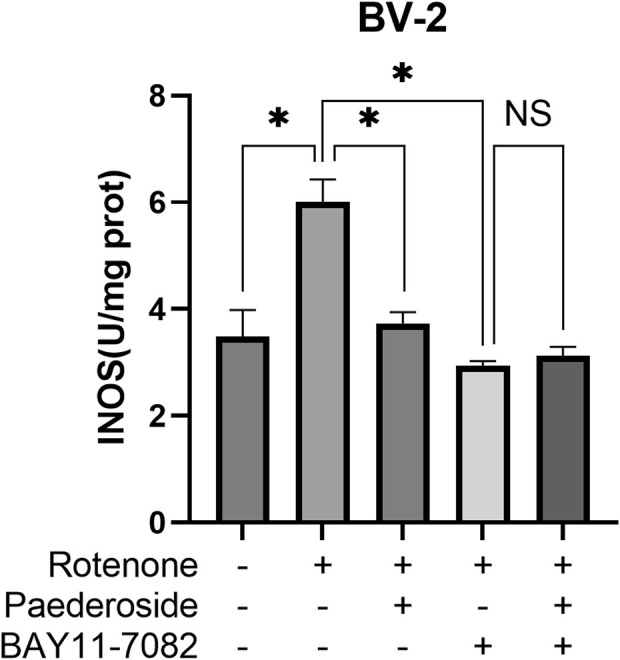
Effect of paederoside on iNOS activities in rotenone-induced BV-2 microglial cells under NF-κB pathway inhibition by BAY11-7082. (n = 3). *P < 0.05, ^NS^P > 0.05.

As shown in Figure 9, the control group exhibited iNOS activity of 3.48 ± 0.50 U/mg protein, whereas rotenone treatment significantly increased this value to 6.01 ± 0.42 U/mg protein, representing a 72.7% increase relative to baseline values (P < 0.05). Paederoside treatment effectively reduced iNOS activity from 6.01 ± 0.42 U/mg protein in the model group to 3.73 ± 0.21 U/mg protein, reflecting a 37.9% decrease (P < 0.05). Subsequent treatment with the NF-κB inhibitor BAY11-7082 further diminished iNOS activity to 2.94 ± 0.08 U/mg protein in the model group (P < 0.05). When BAY11-7082 was introduced 2 h prior to rotenone exposure, no significant difference was observed between groups treated with and without paederoside (3.12 ± 0.17 U/mg protein versus 2.94 ± 0.08 U/mg protein, P > 0.05), indicating that paederoside’s inhibitory effect on iNOS is mediated through the NF-κB pathway.

### 3.8 Effect of paederoside on α-syn nitration triggered by rotenone in an N2A/BV-2 co-culture model

**FIGURE 10:**
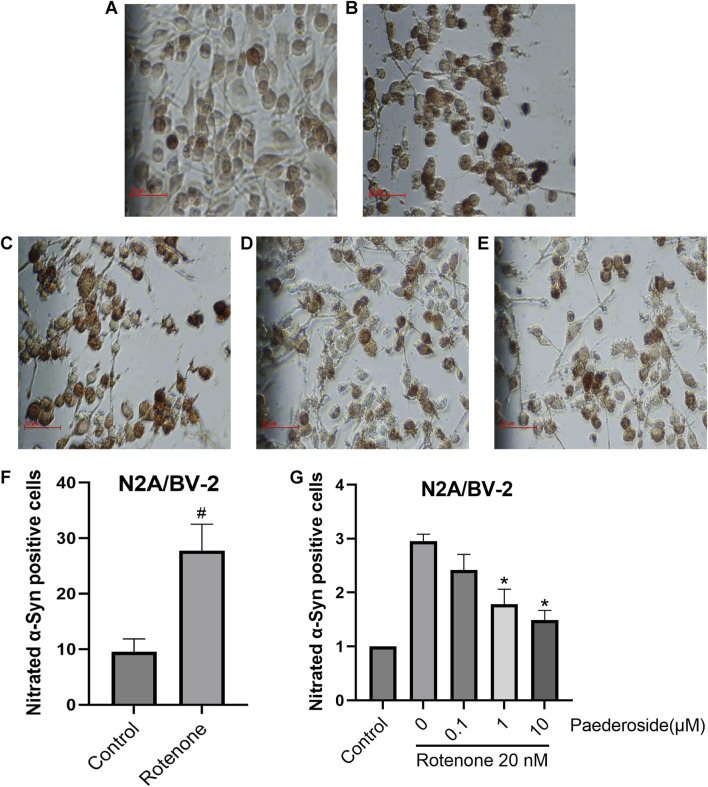
Effect of paederoside on α-Syn nitration triggered by rotenone in an N2A/BV-2 co-culture model (n = 3). **(A)** Conrol group; **(B)** Rotenone group; **(C)** Rotenone+0.1 micromolar (μM Paederoside; **(D)** Rotenone+1 μM Paederoside; **(E)** Rotenone+10 μM Paederoside; **(F)** 20 nM rotenone treatment resulted in a significant increase in nitrated α-Syn in the N2A/BV-2 cell co-culture model; **(G)** Paederoside significantly reduced the levels of nitrated α-Syn induced by rotenone in an N2A/BV-2 co-culture model. *P < 0.05 as compared with rotenone-treated group and ^#^P < 0.05 as compared with control group.

As shown in Figure 10F, the control group exhibited 9.52 ± 1.17 nitrated α-Syn-positive cells, while the rotenone-exposed group showed a significant increase to 27.74 ± 2.38 (P < 0.05), indicating a nearly threefold increase relative to controls. In Figure 10G, with the control group standardized to 1.00, the model group presented 2.95 ± 0.13 nitrated α-Syn-positive cells. Paederoside treatment at 1 μM and 10 μM significantly attenuated rotenone-induced nitration, yielding normalized cell counts of 1.78 ± 0.28 and 1.49 ± 0.18 (both P < 0.05). However, 0.1 μM paederoside did not produce a statistically significant change on α-Syn nitration (2.42 ± 0.29 versus 2.95 ± 0.13, P > 0.05).

### 3.9 Protective effect of paederoside on rotenone-induced injury in N2A cells

**FIGURE 11:**
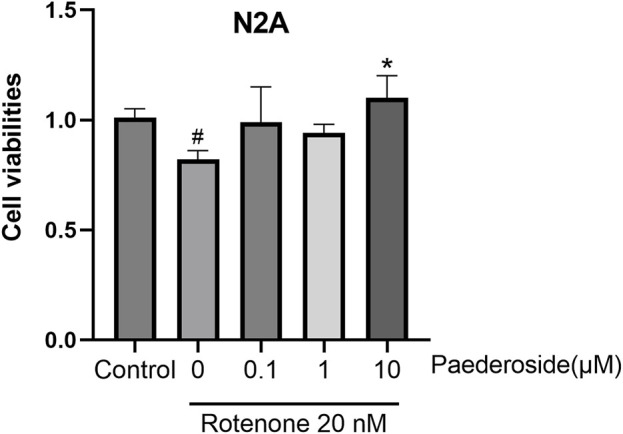
3.8 Protective effect of paederoside on rotenone-induced injury in N2A cells (n = 3). *P < 0.05 as compared with rotenone-treated group.

As shown in Figure 11, N2A cells in the control group exhibited a survival rate of 1.01 ± 0.02. When subjected to 20 nM rotenone-induced damage, cell viability reduced to 0.82 ± 0.02 (P < 0.05). Pretreatment with 10 μM paederoside markedly enhanced N2A cell survival to 1.10 ± 0.05 (P < 0.05). In contrast, lower concentrations of 0.1 μM and 1 μM paederoside resulted in survival rates of 0.99 ± 0.08 and 0.94 ± 0.02, respectively, showing no statistically significant improvement compared to the model group.

## 4 Discussion

The prolonged accumulation of misfolded protein aggregates—known to impair mitochondrial function, disrupt synaptic integrity, and reduce neuronal viability—suggests that comprehensive analysis of RNS-induced protein aggregation processes may reveal viable biochemical targets for pharmacological intervention in neurodegenerative disorders (Nakamura and Lipton, 2008). Our experimental findings demonstrate that paederoside exerts neuroprotective properties in rotenone-exposed neuronal cultures by suppressing NO production and modulating the NF-κB/NOS/NO/α-Syn nitration signaling pathway.

In this study, N2A, BV-2 cells, and N2A/BV-2 co-culture *in vitro* were utilized to investigate the protective effects of paederoside on cell models of PD induced by rotenone. N2A has been extensively utilized to investigate neuronal differentiation, axonal growth, and signaling pathways (Tremblay et al., 2010). N2A cells express Nurr-related factor 1 and exhibit limited expression of TH along with low levels of dopamine. The presence of db-cAMP significantly enhanced both TH expression and dopamine levels, as demonstrated by Western blot analysis, immunocytochemistry, and high-performance liquid chromatography (Tremblay et al., 2010).

*In vitro* models provide advantages over *in vivo* models, including lower costs, reduced complexity, controlled environments, and high-throughput screening capabilities. However, they have limitations as they lack the complex interactions of whole organisms and do not fully replicate disease progression (El Elhaj and Onger, 2025). The rotenone model is particularly suitable for exploring the role of mitochondrial dysfunction and Lewy body formation in the substantia nigra, which constitutes a hallmark pathological feature of PD (Zhang et al., 2022). The pathophysiological causes associated with PD include oxidative stress, mitochondrial dysfunction, inflammation, abnormalα-Syn, and endogenous neurotoxins. These elements suggest the presence of a detrimental cycle (Sun et al., 2019). A substantial body of evidence has demonstrated that pathologicalα-Syn and its aggregates play a pivotal role in the pathogenesis of PD (Hideshima et al., 2022). Pathological aggregation ofα-Syn is a key mechanism in PD, causing mitochondrial dysfunction, oxidative stress, and the degeneration of dopaminergic neurons (Gao et al., 2025). The temporal and spatial development of oligodendrocytic α-Syn pathology in conjunction with neuronal pathology in PD (Otero-Jimenez et al., 2025). α-Syn exerts its effects at the postsynaptic level by interacting with specific subunits of the glutamate N-methyl-D-aspartate receptor, thereby modulating corticostriatal plasticity in distinct neuronal populations (Marino et al., 2022).

Paederoside, a bioactive compound derived from *P*. *scandens* (Lour.) and *P*. *foetida*, exhibits notable medicinal value. *P. scanden*s, a member of the Rubiaceae family, is widely distributed across southern Korea, Vietnam, India, China, Japan, the Philippines, and the United States (Trung et al., 2023). Its congener *P. foetida* is also pharmacologically important within the genus (Wang et al., 2014). In Traditional Chinese Medicine, these plants have been historically used to manage rheumatoid arthritis, bacillary dysentery, dental pain, thoracic discomfort, and hemorrhoidal conditions (Xu et al., 2023).

Modern studies confirm the diverse bioactivities of *Paederia* species, including oxidative stress mitigation, inflammation modulation, microbial inhibition, tumor suppression, and antidiarrheal effects (Ai et al., 2023; Wu et al., 2019). (Mhyre et al., 2012) Oxidative stress mitigation: Oxidative mechanisms are key contributors to neurodegenerative pathologies, making antioxidant interventions clinically significant (Aborode et al., 2022). Essential oil of *P*. *scandens* has demonstrated notable antioxidant activity by downregulating heat shock cognate 71 kDa protein (Wu et al., 2019). Similarly, *P. foetida* leaf extracts reduce lipid peroxidation and enhance superoxide dismutase (SOD), glutathione peroxidase, and catalase (CAT) activities in diabetic rodent models (Kumar et al., 2014). In hepatic protective studies, total iridoid glycosides from *P. scandens* significantly attenuated carbon tetrachloride (CCl_4_)-induced oxidative damage in rats, evidenced by increased levels of glutathione (GSH)/CAT/SOD, alongside decreased malondialdehyde (MDA) concentrations (P et al., 2015). (Percario et al., 2020) Inflammation regulation: The aqueous fraction of *P. scandens*’ methanolic extract has demonstrated analgesic activity in both chemically induced and thermal nociception, potentially mediated by iridoid glycosides and polysaccharides (Chu et al., 2008). Mechanistic studies have indicated that *P. scandens* suppresses Janus kinase 2–signal transducer and activator of transcription 3 phosphorylation, suggesting its therapeutic potential for rheumatoid arthritis via inflammatory pathway inhibition (Chen et al., 2022). Further research has demonstrated that *P. scandens* extracts modulate synovial inflammatory mediators and disrupt NF-κB signaling cascades (Ma et al., 2009). The hepatoprotective effects of this plant against acetaminophen-induced toxicity are likely linked to the iNOS pathway (Tang et al., 2022), while iridoid–lysine interactions inhibit lipoxygenase activity (Ling et al., 2003). (Dong-Chen et al., 2023) Gastrointestinal effects: The n-butanol-soluble fraction of *P. scandens* exhibits antidiarrheal activity via the modulation of the phosphatidylinositide 3-kinase–protein kinase B–NF-κB pathway. Phytochemical analyses have identified paederosidic acid derivatives as likely bioactive constituents responsible for these therapeutic effects (Ai et al., 2023).

IGPS represent a primary class of bioactive constituents extracted from the traditional Chinese medicinal plant *P. scandens* (Lour.) Merrill (Rubiaceae) (Liu et al., 2012). (Mhyre et al., 2012) Administration of IGPS at 70, 140, and 280 mg/kg led to a dose-dependent reduction of mechanical hypersensitivity in spared nerve injury models, as indicated by elevated mechanical withdrawal thresholds. Concurrently, these treatments substantially reduced NOS enzymatic activity and NO and cGMP concentrations while effectively suppressing iNOS mRNA transcription (Liu et al., 2012). (Percario et al., 2020) Experimental evidence indicates that IGPS mitigates renal impairment in UAN rodent models through anti-inflammatory and immunoregulatory mechanisms, mediated via inhibition of NF-κBp65 pathway activation (Zhu et al., 2012). (Dong-Chen et al., 2023) Studies on acute hepatic injury models induced by intraperitoneal CCl_4_ administration demonstrate that total iridoid glycosides from *P. scandens* var. *tomentosa* exert hepatoprotective effects by reduction of oxidative stress. These effects are characterized by elevated levels of GSH, glutathione-S-transferase, and SOD, along with reduced MDA concentrations in hepatic tissues (P et al., 2015).

Recent studies have identified numerous dimeric and monomeric iridoid glycosides—including paederoside and paederosidic acid derivatives—extracted from *P. scandens*. These phytochemicals exhibit diverse biological effects, including antitumor and anti-inflammatory properties (Chen et al., 2014). Research has shown that paederosidic acid binds directly to the P2Y14 receptor, stabilizing its thermal properties and reducing susceptibility to proteolytic degradation. This interaction significantly suppresses osteoclastogenesis mediated by receptor activator of nuclear factor-κB ligand in P2Y14R-deficient rodent models (Li et al., 2023). Furthermore, paederosidic acid has been shown to promote osteogenic activity under lipopolysaccharide-induced conditions through dual mechanisms: mitigating inflammatory responses and oxidative stress by elevating SOD2 activity while simultaneously downregulating tumor necrosis factor-α (TNF-α) expression (Tao et al., 2024).

Experimental findings demonstrate that paederoside effectively reduces nitrated α-Syn levels in rotenone-exposed N2A/BV-2 co-culture models. Innovative therapeutic strategies targeting pathological α-Syn species offer important avenues for future research (Park et al., 2025). The 140-amino acid α-Syn, a conserved presynaptic protein, serves as a primary neuropathological hallmark of PD (He et al., 2021). Core α-synucleinopathies include PD, LB dementia, and multisystem atrophy, with secondary involvement reported in Alzheimer’s disease and related disorders (Kim et al., 2014). Clinical evidence indicates that PD is characterized by widespread α-Syn pathology affecting both central and peripheral nervous systems (Ye et al., 2023). This soluble protein exhibits an inherent tendency to aggregate under certain cellular conditions (Beyer and Ariza, 2013), a process exacerbated by biochemical modifications such as phosphorylation, ubiquitination, nitration, and proteolytic cleavage (Beyer and Ariza, 2013). Pathogenic α-Syn oligomers trigger neuronal damage through multiple mechanisms: mitochondrial dysfunction, endoplasmic reticulum stress, proteostasis impairment, synaptic failure, programmed cell death, and inflammatory responses (Du et al., 2020). Various post-translational modifications (PTMs), including phosphorylation, nitration, acetylation, O-GlcNAcylation, glycation, SUMOylation, ubiquitination, and C-terminal truncation, critically influence α-Syn aggregation kinetics, solubility, degradation, and membrane interactions (Brembati et al., 2023). Neuropathological investigations confirm the presence of modified α-Syn species in PD brains, particularly phosphorylated (Ser129) and nitrated variants within LB inclusions (McCormack et al., 2012). Nitroxidative stress drives these biochemical modifications (Chavarria and Souza, 2013), establishing a direct link to neurodegeneration (He et al., 2019). RNS induce α-Syn alterations that impede fibrillization, producing protease-resistant aggregates with elevated cytotoxic potential (Ischiropoulos, 2003). In neuronal environments, glycation and glycosylation markedly influence α-Syn structure, oligomerization dynamics, and nucleic acid interactions. Glycated forms exacerbate genomic instability through DNA binding and ROS generation during glycation (Guerrero et al., 2013). Dose- and time-dependent neurotoxicity occurs in SH-SY5Y neuroblastoma cells exposed to nitrated α-Syn oligomers (Liu et al., 2011). Longitudinal studies identify phosphorylation at Ser129 (pS129) as a primary early-stage PTM in PD progression, followed by nitration at Tyr39 (nY39), with Ser87 phosphorylation (pSer87) appearing in later stages (Sonustun et al., 2022).

The involvement of dysregulated immune pathways in PD has been extensively documented, with neuroinflammatory processes and microglial activation recognized as key contributors (Fahmy et al., 2019). These neuroinflammatory mechanisms play a pivotal role in the degeneration of dopaminergic neurons characteristic of PD (Reynolds et al., 2008a). The inflammatory actions of microglia significantly influence the progression of nigrostriatal degeneration observed in PD (Reynolds et al., 2009a). Upon exposure to nitrated α-Syn, microglia secrete a combination of neurotoxic and neuroprotective mediators (Reynolds et al., 2008b). These responses are partially initiated by misfolded nitrated α-Syn derived from LBs, which are released by degenerating or deceased dopaminergic neurons (Reynolds et al., 2009a). Nitrotyrosine modifications of α-Syn may impair immune tolerance and activate peripheral leukocytes in lymphoid tissues, triggering adaptive immune reactions that exacerbate PD pathogenesis (Ben et al., 2008). Under oxidative conditions, α-Syn undergoes nitration and, when aggregated, enhances pro-inflammatory microglial activity (Reynolds et al., 2008a).

NOS can be categorized into constitutive isoforms (eNOS, nNOS) and inducible isoforms (iNOS). iNOS is predominantly expressed in intracranial astrocytes and microglia, whereas neuronal nitric oxide synthase (nNOS) is primarily found in neurons. We employed BV-2 cells, a microglial cell line, to evaluate the effect of paederoside on iNOS expression in rotenone-induced cellular models. Only BV-2 microglial cells were selected for this investigation; N2A neuronal cells were not included in the study of iNOS. SMT, a selective inhibitor of iNOS, was administered exclusively in the BV-2 microglial model and not in the N2A neuronal model. Emerging research demonstrates that suppressing NOS significantly reduces L-3,4-dihydroxyphenylalanine-triggered dyskinesia in rodent models (Del-Bel et al., 2011). Behavioral deficits and reductions in tyrosine hydroxylase-positive neurons observed after short-term rotenone exposure correlate with elevated NO levels in rodent neural tissues (Xiong et al., 2015). Increased NO production, mediated by neuronal NOS or microglial-inducible NOS, contributes substantially to 1-methyl-4-phenyl-1,2,3,6-tetrahydropyridine-induced neurotoxicity (Tieu et al., 2003). Co-administration of nanodelivered neuronal NOS and phosphorylated tau antibodies with cerebrolysin notably attenuated Parkinsonian neuropathology exacerbated by closed-head injury (Ozkizilcik et al., 2023). Both iNOS activation and focal adhesion kinase suppression appear to be involved in cellular degeneration triggered by nitrated α-Syn (Liu et al., 2011). Pathologically modified, nitrosylated α-Syn aggregates from degenerating dopaminergic neurons diffuse into the extracellular space, where specific CD4^+^ lymphocyte populations modulate microglial immune responses to these aberrant proteins (Reynolds et al., 2009b). Nitrosylation induces structural reorganization characterized by enhanced secondary structure and promotes neutral pH-dependent oligomer formation compared to native disordered monomers (Uversky et al., 2005). PTMs of α-Syn represent promising therapeutic targets for treating α-synucleinopathies (Brembati et al., 2023).

To investigate whether paederoside exerts anti-PD effects by modulating NOS activity via NF-κB, two experimental studies were conducted. First, we examined whether paederoside influences NO production—the enzymatic product of NOS—through the inhibition of NF-κB, as illustrated in Figures 8A,B. Second, we assessed the effect of paederoside on NOS enzyme activity under NF-κB inhibition, as shown in Figure 9. In the presence of the NF-κB inhibitor BAY11-7082, rotenone-induced NO production was markedly attenuated in BV-2 microglial cells. This pharmacological intervention demonstrates that NF-κB signaling plays a pivotal regulatory role in mediating inflammatory responses via NO modulation. Pretreatment with BAY11-7082 effectively suppressed rotenone-induced NF-κB nuclear translocation, subsequently reducing the transcriptional activation of pro-inflammatory mediators, including TNF-α and interleukin-6. These results indicate that the neuroinflammatory effects induced by rotenone are mediated through the NF-κB signaling cascade and that targeted inhibition of this pathway may mitigate inflammation-associated neurotoxicity in microglial activation models. The addition of a cell line model would further substantiate the impact of paederoside on NOS activity and enhance the validity of our findings.

To illustrate the protective effects of paederoside on cell models of PD, two experiments were conducted. First, to evaluate the impact of paederoside on TH-positive cells within the N2A/BV-2 co-culture, as shown in Figure 10. Second, to assess the effect of paederoside on the viability of N2A cells *in vitro*, as presented in Figure 11. Given that BV-2 cells are of microglial origin, no experimental conditions were designed to investigate the potential effects of paederoside on BV-2 cell activity.

In summary, *in vitro* data suggest that targeting the NF-κB/NOS/NO/α-Syn nitration pathway represents a promising therapeutic strategy for addressing PD. Furthermore, the study demonstrates that the plant-derived bioactive compound paederoside exhibits neuroprotective effects by modulating this molecular pathway. However, PD is characterized by progressive, region-specific neurodegeneration, non-cell autonomous mechanisms, and the interplay of factors such as aging, neuroimmune interactions, and systemic metabolic processes. In future research, *in vivo* models or human-derived dopaminergic neurons will be employed to enhance the validity of our translational assertions.

## Data Availability

The raw data supporting the conclusions of this article will be made available by the authors, without undue reservation.
